# Competition and efficiency in repeated procurements: Lessons from the Finnish rehabilitation markets

**DOI:** 10.1002/hec.4485

**Published:** 2022-02-20

**Authors:** Visa Pitkänen

**Affiliations:** ^1^ Research Department Social Insurance Institution of Finland Helsinki Finland

**Keywords:** competition, competitive bidding, health care, price, public procurement

## Abstract

Inefficient practices and lack of competition are common problems in public procurements. In this study, I examine the effects of a procurement practice reform in the Finnish rehabilitation markets where providers are acquired in a repeated manner through competitive bidding scoring auctions. Until recently, the largest public procurer did not use any systematic criteria for accepting providers, and only a few providers did not receive a contract. After the reform, providers were systematically accepted based on their capacity and the local demand. I analyze the effects of the reform on prices in physio, speech and occupational therapy services with data that covers five subsequent procurements. I use the pre‐reform differences in local competition within the markets in a difference‐in‐differences setting. The descriptive evidence shows that the reform slowed down the rapid increase of prices in all three services. The regression analysis indicates that effects are strongest in the most competitive local physiotherapy markets. This suggests that increasing entry and competition in the less competitive services and local markets would benefit the public procurer.

## 1 INTRODUCTION

Thousands of public authorities in the EU spend annually around 14% of GDP on the purchase of services, works and supplies from private companies (European Commission, 2020). Typically, publicly financed services are purchased from private providers in order to exploit market competition as an instrument for improving efficiency (Poutvaara & Jordahl, 2020). The success of public procurements depends greatly on the following two factors: efficient procurement practice and a competitive underlying market. However, studies have found that a variety of inefficient practices are common in public procurements (Hyytinen et al., 2018) and they often lack the necessary competition (Jääskeläinen & Tukiainen, 2019). Many publicly financed health and social services are also acquired from private providers through public procurement mechanisms such as competitive biddings (EXPH, 2021). Even though different competitive measures are increasingly used to achieve cost savings in health services, the empirical literature on public procurements in health care is still very scarce.

In this study, I examine the importance of an efficient procurement practice and market competition in the Finnish rehabilitation services, where the largest public purchaser acquires private providers through repeated competitive bidding scoring auctions arranged every 4 years. In particular, I analyze the effects of a procurement practice reform in 2018, which aimed to increase efficiency and price competition. Prior to the reform, the insurance districts that organized the procurements ranked providers based on their price and quality, but did not use any systematic acceptance criteria. In practice, the districts offered a contract to around 99% of all bidders. The reform introduced the following three changes: First, ranked providers were accepted based on their capacity and the local demand for the service. Second, quality weight decreased from the previous 50%–20%, and price weight increased correspondingly. Third, the procurement procedure was centralized from the districts to a specialized procurement department, which increased competence and decreased discretion in the procurement process. The aim of the reform was to increase price competition by increasing bidders' risk of not receiving a contract, while securing the availability of the services throughout the country.

I analyze the price effects of the reform in physio, speech and occupational therapy markets. The procurements were arranged in a similar manner across all three services throughout the country. However, there are notable differences in the level of competitiveness between and within the three markets. Physiotherapy markets are one of the most competitive health care markets in Finland, whereas speech and occupational therapy providers' capacity fails to meet demand in nearly all parts of the country. Therefore, it is very likely that the reform had different effects on providers' bidding behavior depending on the service and on local market competitiveness. I utilize procurement data that includes providers' prices, quality and capacity offers from five subsequent rounds of competitive biddings in all three services. In the analysis, I follow the approach by Propper et al. (2008), and use a difference‐in‐differences setting that exploits pre‐reform geographical variation in competition within the three service markets.

The descriptive evidence shows that the trends showing consistent growth in average prices and average price changes were reversed to a significant degree across all three services in the 2018 procurement. For example, practically all providers in all three services increased their prices in every procurement round prior to 2018. However, in 2018, most providers increased their prices only a little and many providers even decreased their prices compared to the previous procurement. The regression analysis shows that the reform had the greatest impact in the most competitive local physiotherapy markets. However, a similar difference between more and less competitive markets is not present in the speech and occupational therapy markets. These results illustrate that a successful procurement requires an efficient procurement practice and a sufficient level of market competition.

## 2 RELATED LITERATURE

This study connects to several different strands of literature on public procurement and health economics. First, the study contributes to the growing empirical literature on efficient procurement practices, including effects of more competent officials and lower discretion in the procurements. Tas (2020) finds that a high‐quality procurement practice increased the number of bidders and the probability that prices are lower than originally estimated in European public procurements. Bandiera et al. (2009) find that some Italian public purchasers pay systematically more for standardized goods, and that these differences are explained by inefficient purchasing procedures. Hyytinen et al. (2018) show that a change from discretionary beauty contests to a more rule‐based procurement environment resulted in cost savings in Swedish cleaning service procurements. Cameron (2000) finds that limiting purchaser's discretion reduced prices in the US electricity markets, and Coviello et al. (2018) show that greater discretion causes a significant increase in the probability that Italian purchasers will contract the same bidders repeatedly. Decarolis et al. (2020) show that an increase in US federal procurement officials' competence decreased expenditure, time delays and number of renegotiations. Bucciol et al. (2020) also show the importance of competence and discretion in their study on procurements of medical devices in Italy.

Second, the study contributes to the literature on competition in public procurements. The standard auction theory and everyday intuition say that competition is an important requirement for a successful public procurement that produces high‐quality services at reasonable prices (Bajari et al., 2008; Bulow & Klemperer, 1996; Wilson, 1977). In a closely related context, Jääskeläinen and Tukiainen (2019) find that competition is relatively low in Finnish public procurements with a median bidder count of only two.^1^ They also show that a higher number of potential and actual bidders decreases price measures such as win margins. However, this result may not hold if the “common values effect”, “affiliation effect” or “entry effect” dominates the “competition effect” (see Jääskeläinen & Tukiainen, 2019). In short, when bidding is costly (in terms of acquiring information, bid preparation and opportunity costs) and participation in the procurement is endogenous, increasing competition beyond a certain number of bidders may have adverse effects on expected prices (Onur & Tas, 2019). These adverse effects of increased competition on prices have also been found in empirical studies (see Hong & Shum, 2002; Li & Zheng, 2009; Pinkse & Tan, 2005).

Third, the study contributes to the literature on public procurements in health services. The empirical literature is rather scarce and comes mainly from the US. Song et al. (2012, 2013) analyze the Medicare Advantage program, which implemented a competitive bidding system to determine plan payments in 2006. They show that insurers are able to use their market power to obtain higher prices in the procurements. A recent study by Ferraresi et al. (2021) shows that the introduction of centralized procurement within the regional health care systems reduced per capita health expenditures in Italy. Even though competitive measures are increasingly used to achieve cost savings in European health care systems, very little is still known about the nature of procurements in publicly financed health services. This study fills this gap and is the first to analyze the price effects of enhanced competition and procurement practice in repeated procurements for publicly financed health services.

Health service purchasers often select multiple providers simultaneously for a certain contract period in a geographical area (Barros et al., 2016). This has raised such questions as how many providers should be contracted to maintain a competitive market environment (McCombs & Christianson, 1987). Hoerger and Waters (1993) present a theoretical model of providers' behavior in markets where providers first participate in a competitive bidding and then compete for patients with other selected providers. They show that selecting multiple providers in the procurement may enhance price and quality competition. This structure is present also in the studied markets, which combines competition *for* the market (procurement) and *in* the market (patient choice).^2^

Quality is often a key interest for procurers, patients and other stakeholders in health care. Public purchasers typically use scoring auctions, where providers compete for the market on both price and quality, and the “economically most advantageous bidders” are contracted. Several studies offer theoretical views on the properties of scoring auctions compared to other mechanisms such as first‐price auctions, so‐called beauty contests and direct bargaining (see Asker & Cantillon, 2008, 2010; Bergman & Lundberg, 2013; Branco, 1997; Che, 1993).

Various long‐term care services share characteristics similar to the studied rehabilitation services. Van Eijkel et al. (2018) provide evidence from procurements of daily housekeeping services by the Dutch municipalities. Their results show that higher competition has a moderate negative effect on prices. Forder and Allan (2014) study the English care home markets, where local authorities negotiate prices with providers, and find that greater competition reduces prices. Studies from the US nursing home markets also show that competition has a small negative effect on prices (Mukamel & Spector, 2002; Nyman, 1994).

Finally, this study directly continues the previous work by Pekola et al. (2017) and Pitkänen et al. (2020). Pekola et al. (2017) analyze the effects of competition on prices in the physiotherapy market using a sample of providers from the 2010 procurement, finding that greater competition had no effect on prices. Pitkänen et al. (2020) also examine the physiotherapy procurements using data from 2006, 2010 and 2014. They find that an inefficient procurement practice led to notable price increases as providers learned that the risk of not receiving a contract was very low. They also show that a systematic acceptance rule, similar to what was implemented in the procurement reform, would result in a trade‐off between cost savings and service continuity at patients' usual providers.

## 3 INSTITUTIONAL CONTEXT AND DATA

### 3.1 Rehabilitation services

This study examines competitive biddings for intensive medical rehabilitation services in Finland. These services are financed and organized by the Social Insurance Institution of Finland (Kela), which is the single largest organizer of rehabilitation services in the country. The study focuses in outpatient physio, speech and occupational therapy markets, which are the three largest therapies of individual rehabilitation. All of these services are intended for persons under 65 years of age with disabilities, who face problems managing daily activities and fulfill the criteria defined by the law. The services are provided based on a written rehabilitation plan drawn up with a physician for one to 3 years at a time. Patients do not pay anything out of pocket and have free choice among the accepted providers. Typical therapy sessions last 45 or 60 min and are scheduled a few times a week, depending on each patient's individual rehabilitation plan. Some patients may receive the therapy for several years. The services are organized either at the provider's facilities or remotely in a familiar environment such as the patient's home, school or kindergarten.

Kela organized and financed these three services for a total of 38,762 patients at a total cost outlay of 159.3 million euros in 2018. Figure A1 (in the Appendix) present the trends in the number of patients and in annual nominal costs of the services in 2002–2020. Physiotherapy is the largest service, but there was a particularly large increase in recent years (before the Covid–19 pandemic) in the number of patients undergoing speech or occupational therapy.^3^ The majority of patients in speech and occupational therapies are children who only receive the service for a few years, whereas physiotherapy is received more commonly by patients of all ages, and the rehabilitation typically lasts for many years. Very few patients receive two or more therapies simultaneously.

### 3.2 Competitive biddings

Kela's insurance districts are responsible for organizing the three services for their local populations.^4^ The districts acquire the services from private providers using a competitive bidding scoring auction, which they have organized in a similar and predefined manner in all three services repeatedly since2003.^5^ This study focuses on the competitive biddings that were organized simultaneously in all three services in 2003, 2006, 2010, 2014 and 2018. The procurement process has been as follows: Kela publishes a request for tenders, after which the providers give information on their quality and annual capacity and set a price for a 45‐min rehabilitation session in their sealed bids. The bids are submitted to the district in which providers are based or in which they wish to operate remotely. The districts evaluate the tenders and rank the providers that meet the minimum criteria based on their quality‐price scores. Each district decides an acceptance threshold, which should be based on the estimated demand for the service and the capacity of the providers. Providers above the threshold are offered a contract and providers that sign the contract form the pool of providers. Providers are paid for patients' visits based on their own accepted price bid.^6^ Since 2006, the districts have sent the quality‐price rank lists to each provider that submitted a tender in that district, which has enabled providers to evaluate their tenders afterwards.^7^

However, prior to 2018, the districts did not apply systematic acceptance criteria or rules in the competitive bidding, and the most common acceptance threshold was below the provider ranked last. Therefore, very few providers that fulfilled the minimum requirements failed to receive a contract offer in each of the three services. For example, out of 25 districts in the 2010 procurement, only three rejected at least one provider in physiotherapy and occupational therapy services, and only two in speech therapy service. A previous study (Pitkänen et al., 2020) showed that this increased the overall price‐level of the service, because firms eventually learned that the risk of not receiving a contract was very low and that they were able to bid for higher prices. The main reasons for this inefficient practice was that the district officials were not trained procurement experts and did not have strict budget constraints. They also wanted to enable patients to choose from a large pool of providers and to avoid the possible negative feedback that could have resulted from several patients being forced to leave their usual provider.

Kela made two notable public changes, and one internal practical change, to its procurement practices in 2018. First and foremost, Kela introduced systematic acceptance criteria based on providers' capacity and the current number of patients in each insurance district. All providers were accepted for inclusion on the quality‐price rank list until the required capacity in the area was covered, and some additional providers were allowed in based on their location and language skills. In practice, providers were required to list all municipalities to which they would provide the service, and, in each municipality, at least three providers were accepted for physiotherapy and one provider for speech and occupational therapies. Some providers were accepted also in larger cities to ensure that local demand for the service would be fulfilled. In addition, many Swedish‐speaking providers were accepted in the western and southern districts.^8^ All accepted providers were offered a contract for 1 year with options for three additional years.

Second, Kela decreased the quality weight to 20% and increased the price weight to 80% in 2018. Previously, the quality‐price scoring rule was a simple quality/price assessment in 2006, which changed to quality being weighted at 60% and price at 40% in 2010, and both weighted at 50% in 2014. Although the quality weight decreased, the minimum quality standards to which all providers need to adhere were increased. In general, quality is difficult to measure in health care because it is multidimensional (Tay, 2003). Quality measures are often based on rough measurements such as mortality, or inputs such as the number of beds. In all studied Kela procurements, the main quality categories have been the following process indicators: providers' experience with disabled patients, additional education and investments into facilities. Equipment has also been a category in the physiotherapy procurements.

In this study, the potential effects of changing the quality and price weights cannot be separated from the overall effects of the procurement reform. In the short term, it is very likely that the reform had a greater effect on prices than on quality, because the quality measures are investments in the provider's physical or human capital. The measures can be seen as a proxy for providers' underlying performance quality or utility gain construct (Forder & Allan, 2014). Increasing (or decreasing) such quality measures usually requires long‐term planning, for example, renting new facilities or hiring new workforce. In this case, providers' only had a few months to react to the new quality weight.^9^ To avoid potential quality reductions during the contract period, Kela requires all providers to commit to maintaining quality at the level of their bid.

Kela also centralized the procurement procedure away from the insurance districts, which did not have much procurement expertise or education, to a specialized procurement department in its central administration. This change separated officials who work with patients from the procurement process, and decreased the discretion related to selecting accepted providers. Based on interviews with Kela's procurement officials, the aim of the reform was to increase price competition, while ensuring the availability of the services throughout the country. The acceptance criteria were specified in the request for tenders, and Kela publicly informed the providers through a variety of channels that not all providers would be offered a contract as had been the case in previous procurements. After the procurement, Kela received a lot of negative feedback from patients, because many of them were forced to leave their usual provider, and from providers, because the quality weight was decreased. Several rejected providers made an official complaint to the Finnish Market Court. The change in procurement practice also gained some attention in the media.

### 3.3 Data

The main data source in this study are providers' quality–price evaluation lists from Kela's insurance districts compiled for the 2006, 2010, 2014 and 2018 procurements, and a list of contracted providers' prices in the 2003 procurement. The quality–price evaluation lists include quality scores, prices for 45‐min service and annual capacities of all providers that fulfilled the minimum quality requirements. The data also includes the number of listed therapists for each provider in 2018. Providers' postal code, business identity code and business type information were collected from Kela's internal provider registry. Patient volumes and costs are retrieved from Kela's statistical database Kelasto. Tables A1–A3 (in the Appendix) present detailed descriptive statistics on accepted and rejected providers' prices, quality and capacity and on price changes in the procurements. Kela piloted beauty contest procurements with fixed prices for physiotherapy services in two insurance districts in 2010. Physiotherapy providers in these areas are therefore not included in the analysis. Providers' quality scores are not comparable between the three services and years, because quality was evaluated using different scales in the procurements.

### 3.4 Market characteristics

There are some notable differences between the markets for the three rehabilitation services. Physiotherapy markets are among the most competitive health services in Finland, both in terms of the number of providers and their capacity: Based on Statistics Finland registries there were a total of 2632 physiotherapy firms in 2015, 1253 of which had a contract with Kela. Kela is the single largest financer of physiotherapy services, accounting for around a quarter of the entire sector's revenue. Unfortunately, similar statistics are unavailable for the speech and occupational therapy markets, which are not nearly as competitive as the physiotherapy market. Based on interviews with providers and Kela officials, speech and occupational therapy firms rely significantly more on the services financed by Kela.^10^ Patients of these two services also may need to wait for available providers or therapists for several weeks or even months in some areas, which reflects the lack of competition and capacity. These differences in level of competition are largely attributable to the supply of the education required to enter these professions, as the annual number of graduating speech and occupational therapists is much smaller than of physiotherapists.^11^

Table 1 presents characteristics of the three rehabilitation markets in the 2006, 2010, 2014 and 2018 procurements. The number of bidders, accepted providers and their capacity, as well as the number of patients are much higher in physiotherapy than in speech or occupational therapy services. The table illustrates how small the number of rejected providers was in 2006, 2010 and 2014, and shows how the reform influenced the share of accepted providers in the three markets: In physiotherapy markets, the number of rejected providers increased from 28 (2.4% of bidders) in 2014 to 295 (27.1%) in 2018. While the number of accepted providers decreased, the total capacity of these providers was still more than double the number of patients. Similar effects are present in the occupational therapy market, where the number of rejected providers also increased significantly from only four (0.1%) in 2014 to 52 (10.1%) in 2018. However, in speech therapy the number of rejected providers increased little from four (0.6%) in 2014 to nine (1.5%) in 2018. These increased rejection rates can be seen as a measure of the overall level of competitiveness in the three markets.

**TABLE 1:** Market characteristics in the procurements

	2006	2010	2014	2018
*Physiotherapy*				
Bidders	1295	1204	1187	1088
Accepted providers	1284	1197	1159	793
Rejected providers	11	7	28	295
Capacity	∼35,000	40,431	47,512	34,988
Patients	13,508	13,067	13,481	14,974
*Speech therapy*				
Bidders	486	488	581	589
Accepted providers	480	481	577	580
Rejected providers	6	7	4	9
Capacity	∼8000	8590	11,990	13,842
Patients	4651	5057	7439	11,757
*Occupational therapy*				
Bidders	330	396	485	517
Accepted providers	325	388	481	465
Rejected providers	5	8	4	52
Capacity	∼8000	10,556	16,168	15,701
Patients	4407	4628	6491	10,793

*Note*: The table includes bidders that fulfilled the minimum requirements. The 2006 numbers on accepted total capacity are estimates based on available data.

Table A4 (in the Appendix) presents additional provider characteristics in the 2018 procurement, and illustrates that there are also some notable differences between providers in the three markets. For example, 91% of physiotherapy firms had their own premises, whereas 83% of occupational therapy providers and only 70% of speech therapy providers had permanent premises. This confirms that remote services are more common in speech and occupational therapies, even though most providers with premises also offer services remotely. Physiotherapy providers are also, on average, larger than speech and occupational therapy providers in terms of their capacity and number of therapists. The majority of providers are independently operating firms, but 30%–40% of providers in all three markets were part of a larger chain. Some of these chains operate regionally, for example, within one district, but some operate nationwide. In principle, all providers are private firms that operate on a for‐profit basis, but some providers may also have altruistic purposes.^12^

## 4 EMPIRICAL APPROACH

Estimating the direct effects of competition on prices in public procurements is challenging due to reverse causality, omitted variables and the selection of bidders via entry (Jääskeläinen & Tukiainen, 2019). The focus of this study is on analyzing the effects of the procurement reform in areas and services that differ in terms of the degree of competition. My empirical approach follows Propper et al. (2008), who analyze the impact of competition on quality in the English National Health Service. They exploit policy reforms in the 1990s and use the fact that the pre‐reform degree of competition differed between geographical areas. This approach has become common in health economics studies that analyze the effects of various competition‐enhancing policies in different contexts (Cooper et al., 2011; Dietrichson et al., 2020; Gaynor et al., 2013; Roos et al., 2020). I use the same approach and exploit the pre‐reform variation in market competition in a difference‐in‐difference (DID, henceforth) setting. The approach is based on the intuitive idea that the reform would have the most impact for providers located in areas with greater market competition, and the least impact in areas with less competition and idle capacity.

I use two different institutional features to define the primary treatment and control groups. First, I account for regional differences in rejections in the pre‐reform procurements, as they already influenced prices prior to 2018 (Pitkänen et al., 2020). Providers that had experienced at least one rejection in their district's quality‐price list can be thought to have prior experience of a slightly more efficient procurement practice. Therefore, the focus of this study is on providers that are located in districts where all providers were offered a contract in the 2006, 2010 and 2014 procurements. Unfortunately, the data does not include information on rejected providers in the first procurement in 2003, but according to a Kela procurement official, the number of rejected providers in 2003 was most likely similar or even smaller compared to 2006.

Second, I use the acceptance criteria of the 2018 procurement. In 2018, Kela accepted at least one bidder per municipality in speech and occupational therapy services, and at least three bidders per municipality in physiotherapy service. This criterion was spelled out in the request for tenders and known to the bidders in 2018, but was not an acceptance criterion in the pre‐reform procurements. Based on these features, the primary treatment group consists of providers that are located in municipalities where all providers were offered a contract prior to 2018 and the number of bidders in 2014 was at least two in speech and occupational therapy services, and at least four in physiotherapy. The control group then consists of providers located in municipalities where all providers were offered a contract prior to 2018 and the number of bidders in 2014 was smaller or the same as mentioned in the acceptance criteria in 2018. In practice, neither providers in the treatment group nor those in the control group had experienced an efficient procurement practice prior to 2018, but the treatment group consists of providers that are located in initially more competitive areas where the reform can be thought to have a greater impact.

The empirical approach divides providers into three groups: the treatment and control groups which both consist of providers that had not faced rejections in their area prior to the reform, and providers that were located in areas with prior rejections. Providers in this third group are excluded from the regression analysis. Table 2 presents descriptive statistics on the three groups and the difference between the treatment and control groups in 2014, that is, prior to the reform. The table shows that the control group includes 271 providers (23% of all providers) in physiotherapy, but consists of only 43 providers (7%) in speech therapy and 54 providers (11%) in occupational therapy. Compared to the control group, providers in the treatment group were naturally located in municipalities with a higher level of competition in all three markets. The two groups had similar prices and capacities in all markets, but the treatment group consisted of higher quality providers in the physiotherapy and occupational therapy markets prior to the reform. In addition, the table also shows that providers located in areas with pre‐reform rejections faced greater competition compared to the treatment and control groups, which means that accounting for previous rejections makes the treatment and control groups more comparable.

**TABLE 2:** Descriptive statistics on the provider groups in 2014

Variable	Treatment				Control				Excluded				Difference (T‐C)	
	Mean	SD	Min	Max	Mean	SD	Min	Max	Mean	SD	Min	Max	Mean	SE
*Physiotherapy*														
Competition	19.53	15.96	4	62	1.98	0.79	1	3	40.85	26.41	2	77	17.55	0.970***
Price (€)	57.42	9.28	34	90	56.45	10.29	38	102.5	60.45	9.94	38.5	116	0.971	0.714
Quality	37.70	7.51	9	53	36.04	7.82	12	55	37.16	7.59	10	53	1.657	0.564**
Capacity	40.65	52.31	2	450	35.96	36.91	2	320	43.16	63.96	1	416	4.693	3.545
N	552				271				364					
*Speech therapy*														
Competition	11.55	11.21	2	58	1	0	1	1	57.36	25.72	1	89	10.55	1.712***
Price (€)	100.77	13.38	69	135	100.49	15.55	70	157	101.78	14.92	58	175	0.280	2.248
Quality	30.84	7.98	0	49	30.79	7.93	13	49	33.71	9.00	7	53	0.051	1.309
Capacity	22.33	24.13	1	180	20.70	26.71	2	160	19.33	18.74	1	160	1.634	4.020
N	271				43				267					
*Occupational therapy*														
Competition	10.38	10.30	2	35	1	0	1	1	25.39	5.92	2	31	9.380	1.404***
Price (€)	74.71	10.70	40	110	71.93	10.23	50	96	74.82	8.91	48	105	2.774	1.568
Quality	35.45	7.99	11	54	32.20	9.64	11	50	37.10	7.45	17	49	3.243	1.217**
Capacity	32.26	40.38	0	280	26.57	24.22	2	120	40.16	52.51	1	400	5.686	5.668
N	311				54				120					

*Note*: The last column shows the difference between treatment and control groups and standard errors for *t*‐tests between their means.

Abbrevition: SE, Standard errors.

*p < 0.05; **p < 0.01; ***p < 0.001.

I use variations of the following provider‐level DID model separately in the three services to analyze the effects of the reform:

(1) $$\text{ln}{\left(P\right)}_{\text{jmt}}=\alpha +\beta \left({\text{Treatment}}_{m}*{\text{Reform}}_{t}\right)+\mu_{j}+\delta_{t}+\gamma_{d}+\varepsilon_{\text{jmt}},$$

where ln(*P*)_jmt_ is a natural logarithm of provider *j*'s price located in municipality *m* in procurement year *t*. Treatment_m_ takes value 1 for providers in the treatment group and 0 for providers in the control group. Reform_t_ takes value 1 if the observation is from the 2018 procurement and 0 if from the pre‐reform procurements prior to 2018. Thus, the interaction Treatment_m_ ∗ Reform_t_ takes value 1 if the observation is from 2018 and the provider belongs to the treatment group, and 0 otherwise. The DID coefficient *β* is the parameter of main interest, measuring the price effect of the reform in the treatment group relative to the control group. Variables $\mu_{j}$, $\delta_{t}$ and $\gamma_{d}$ are provider, year and district fixed effects, and *ε*_jmt_ is an error term. Standard errors are clustered at provider‐level. The main specifications are estimated using data from all five subsequent procurements in 2003, 2006, 2010, 2014 and 2018.

As Roos et al. (2020) discuss, using this empirical approach does not identify the effect of the reform per se, but can be thought capable of identifying the differential effects of the reform between initially more and less competitive areas. Fricke (2017) shows that comparing groups where treatment intensity varies requires the following two identifying assumptions: First, the traditional common trends assumption must hold. Second, the sign of the effect should be the same both in treatment and control groups (Fricke, 2017). Then, following Fricke (2017) and Roos et al. (2020), this approach identifies a lower bound for the effect of the reform in more competitive markets compared with the continuation of the inefficient procurement practice in the same areas.

The definition of the treatment and control groups naturally has some caveats. First, geographical boundaries such as municipalities are often not considered as good local market definitions because they are either too small or large (Dranove & Ody, 2015). However, the acceptance criterion in the 2018 procurement was based on municipalities where providers offer their services, and thus the municipality serves as a natural market definition. Second, the number of providers in a municipality in 2014 does not fully represent the local degree of competition, because providers may travel to neighboring municipalities to offer their services remotely. In practice, providers listed the municipalities where they would offer the services in 2018, whereas the calculated number of bidders in 2014 is based on the providers' physical location. This approach, then may underestimate true competition in municipalities where providers are not physically located but offer remote services.

Finally, even though most providers participate in subsequent procurements, new providers may enter and incumbent providers may exit the markets in between the procurements. For the robustness of the empirical results, it is important that the composition of treatment and control groups remains stable over time. For example, if the number of bidders per municipality has increased after the reform in 2014, the control group could have been directly affected by the reform. Table A5 (in the Appendix) shows the number of providers in the treatment, control and excluded groups in the five subsequent procurements. The table shows that the number of providers in the control group peaked in both speech and occupational therapies in 2018. However, the overall number of bidders has also increased in these two services through the years, and clearer evidence of increased participation in the control municipalities would require additional data from future procurements.

## 5 RESULTS

### 5.1 Descriptive evidence

**FIGURE 1:**
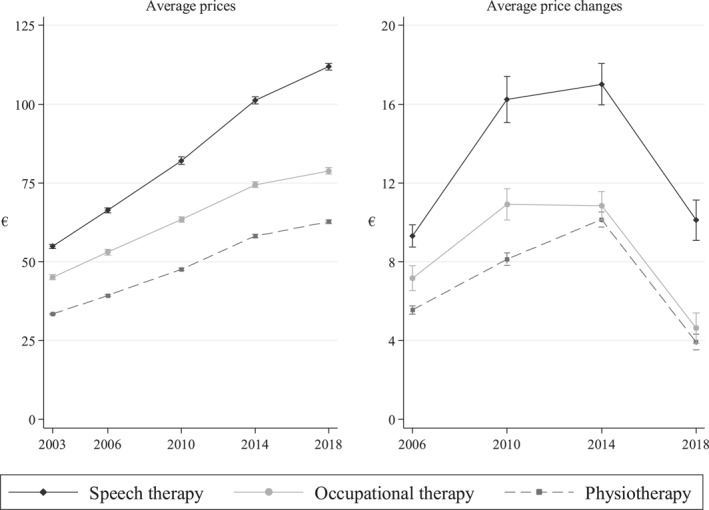
Average prices and price changes in the competitive biddings

Figure 1 presents trends in providers' average prices and price changes in the procurements. The figure illustrates that prices are, on average, highest in speech therapy and lowest in physiotherapy. The average price has increased in all three markets in every procurement. Prices rose particularly rapidly between 2003 and 2014. For example, in physiotherapy the average price rose by as much as 74% from 2003 to 2014. Meanwhile, the Finnish Consumer Price Index increased only by 21% (SVT, 2019). The main reason for these significant price increases was probably that the providers had a low risk of not being included in the pool of accepted providers prior to 2018 (Pitkänen et al., 2020). The figure illustrates the immediate and clear effect that the reform had on prices in 2018: Average prices increased by only 8% in physiotherapy, 10% in speech therapy and 5% in occupational therapy.

Most providers in all three markets have participated in subsequent procurements. For example, 80% of physiotherapy providers in the 2014 procurement had participated also in 2010. The right‐hand side of Figure 1 shows the average price increases in the procurements. The figure shows that the average price changes increased in all three markets in every procurement from 2006 to 2014. Some of the price increases were rather substantial: For example, the single highest price increase was 70 euros by a speech therapy provider in 2014. The effect of the reform is illustrated clearly in 2018, as the average price changes declined sharply across all three markets. The average price increases fell to a similar level as in 2006 in speech therapy, and even more in physiotherapy and occupational therapy. Perhaps surprisingly, the figure shows similar effects in all three markets, even though speech and occupational therapy markets are less competitive than physiotherapy markets.

**FIGURE 2:**
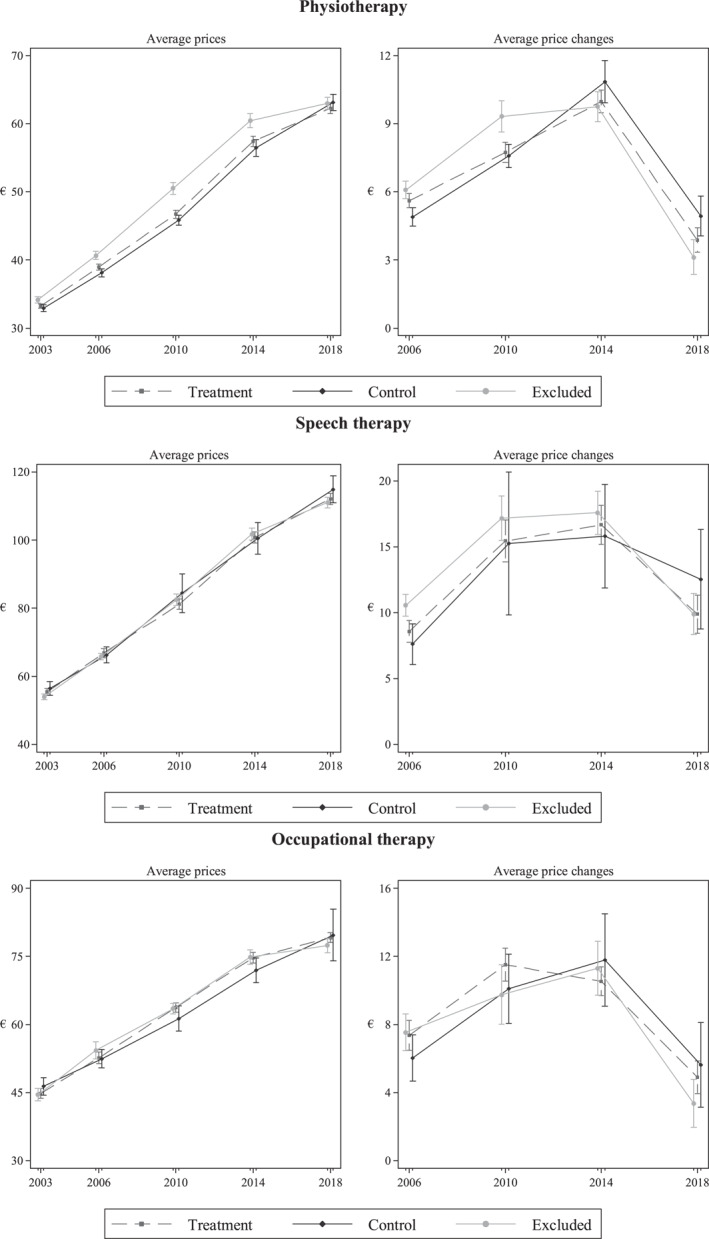
Average prices and price changes in the different provider groups and markets

Figure 2 presents average prices and price changes in the procurements separately for the three groups in all three markets. The left‐hand side of the figure reveals three issues: First, providers that were located in areas with previous rejections had higher prices than the treatment and control groups prior to the 2018 procurement, especially in the physiotherapy market. Second, the figure suggests that the price trends were rather similar in the treatment and control groups in all three services prior to 2018. The figure also confirms that accounting for the previous rejections makes the treatment and control groups' price trends more consistent, especially in the physiotherapy market. Third, the figure suggests that the 2018 reform had the greatest effect on prices in the treatment and rejections groups, as the average prices increased the most in control groups in all three markets. The right‐hand side of the figure shows price changes in the procurements. These figures indicate that providers in the control group increased their prices slightly more than the other two groups in all three markets in 2018, but these differences are statistically insignificant.

More detailed evidence on prices and price changes is presented in the online Appendix. Tables A1–A3 present the descriptive statistics regarding accepted and rejected providers' prices, quality, capacity and price changes in the procurements. The tables confirm that very few providers with a low quality‐price ratio failed to receive a contract offer prior to 2018 in all three services. Importantly, the tables show direct effects of the more efficient procurement practice in 2018 from the procurer's perspective: Accepted providers had significantly lower prices and higher quality than rejected providers in physiotherapy and occupational therapy markets. However, this effect is not present in the speech therapy market, as only nine providers were not offered a contract even in the 2018 procurement.

Figure A2 shows price distributions in the procurements for the three markets. Low price dispersion is perhaps the most common measure of procurement efficiency (Scheffler et al., 2016), even though it can also be a sign of collusion especially if the number of bidders is small. The figures show that price dispersion increased from 2006 to 2014 in all three services, but this trend ended in 2018. Figure A3 shows similar histograms regarding price changes. The effect of the reform is illustrated clearly in these histograms, as 2018 was the first procurement in which several providers decreased their prices in all three markets. In conclusion, all the descriptive evidence suggests that the procurement reform had a clear negative effect on prices.

### 5.2 Regression results

Table 3 shows the results of the main specifications for the three services. The results show that the reform had a greater negative effect on prices in initially more competitive areas in the physiotherapy market. The negative effect of 3.7% is statistically significant and supports the graphical evidence presented in Figure 2. The sign and magnitude of the estimates are very similar also in speech and occupational therapy markets, but these results are statistically insignificant.

**TABLE 3:** Effect of the reform on prices

	Physiotherapy	Speech therapy	Occupational therapy
DID	−0.037***	−0.041	−0.028
	(0.010)	(0.024)	(0.023)
Provider FE	Yes	Yes	Yes
Year FE	Yes	Yes	Yes
District FE	Yes	Yes	Yes
Observations	4298	1362	1518
Providers	1517	571	655
*R* ^2^	0.724	0.721	0.642

*Note*: Dependent variable: Ln(Price). Standard errors (in parentheses) are clustered by provider.

Abbreviations: DID, difference‐in‐differences; FE, fixed effects.

*p < 0.05; **p < 0.01; ***p < 0.001.

The results indicate that especially physiotherapy providers in initially more competitive areas responded to the increased risk of not being included in the pool of providers. There are at least three potential explanations why similar statistically significant effects are not found in the speech and occupational therapy markets. First, the empirical approach offers a lower bound estimate for the effect of the procurement practice reform. Second, the treatment and control groups are small compared to those in the physiotherapy markets, which produces large confidence intervals. For example, the descriptive evidence (see Figure 2) suggests that the effects may have been slightly larger in more competitive areas, but these differences are statistically insignificant unlike in the physiotherapy market. Third, there are differences in the degree and nature of competition in physiotherapy markets compared to the speech and occupational therapy markets. For example, the two markets have less idle capacity from the procurer's perspective, and the services are more often delivered remotely, decreasing the importance of physical premises and local competition.

The negative effect of a little under four percent in the physiotherapy market corresponds to an average price decrease of around 2.3 euros in the treatment group. Because the reform had a smaller but still negative impact on prices also in the control group, and the two groups share similar pre‐trends, the empirical strategy should fulfill the two assumptions discussed by Fricke (2017). Thus, the result can be interpreted as a lower bound for the effect of the reform in these initially more competitive areas. The following simple back‐of‐the‐envelope calculation shows the financial impact of the reform in the physiotherapy market: Municipalities in the treatment group accounted for 7664 patients and costs of 31.5 million euros in 2019, which equals to around 47% of all patients and total costs. An average price increase of 3.7% for contracted providers would then have resulted in around 1.2 million euros higher annual costs in these areas. As noted, these cost savings are only a small part of the overall impact of the reform, because the reform had a negative impact on prices also in the control group, as well as in areas with rejections already prior to 2018.

### 5.3 Robustness checks

Table A6 in the Appendix presents the following robustness checks: The first column offers a formal test for the crucial parallel trends assumption, showing a placebo treatment that takes place in 2010. These results confirm that the treatment and control groups share similar price trends prior to the reform. Similar results are also found if the placebo treatment takes place in 2014. The second column of the table shows results for a model where the treatment and control group definition does not account for previous rejections but only the acceptance criteria in the 2018 procurement. These models suggest that accounting for previous rejections does not influence the regression estimates. However, as shown in the descriptive analysis, accounting for previous rejections makes the treatment and control groups more comparable. The third column of the table adds controls for providers' quality and capacity. These models do not include the years 2003 and 2006, because the data does not contain information on all providers' quality‐scores and capacities from these procurements. Because providers' quality scores are not comparable, I have transformed the quality scores into simple quintiles in each service and year. The results suggest that providers with higher quality offered slightly higher prices in the procurements. In addition, I have estimated models that do not include the first procurement year 2003, as this data may not include all bidders that did not receive a contract. The results for these models also confirm the main results of the study.

The primary treatment group consists of providers that are located in municipalities where the number of bidders in 2014 was at least two in speech and occupational therapy services, and at least four in physiotherapy. Figure A4 presents the price effects of the reform with different thresholds for the treatment group in all three markets. The figure shows that the main results range around the initial cutoffs. The only notable difference is that the negative result becomes insignificant in the physiotherapy market when the treatment group is defined similarly as in speech and occupational therapies. This suggests that local monopolies do not drive the prices to any considerable extent in any of the three markets.

## 6 CONCLUSION

This study provides evidence on the effects of a more efficient procurement practice on providers' prices in Finnish rehabilitation markets in 2018. The reform brought three changes: A more rule‐based acceptance criterion based on capacity and demand in a municipality, a decrease of quality‐weight from 50% to 20%, and the centralization of the process to a specialized procurement unit. Nearly all bidders received a contract under the previous procurement practice, which resulted in rapidly increasing prices (Pitkänen et al., 2020). The aim of the reform was to foster price competition by increasing the providers' risk of not receiving a contract. This study focuses on the effects of the reform in physio, speech and occupational therapy markets, which differ greatly in terms of the degree of market competition. The physiotherapy market is one of the most competitive health care sectors in Finland, but in many parts of the country, there is a lack of especially speech therapy providers. Traditional auction theory suggests that greater competition results in lower prices, which suggests that the reform would have the greatest influence in the most competitive service and local markets. Hence, the empirical approach is based on a comparison between providers that were located in initially more or less competitive areas, but had not experienced rejections in the procurements prior to the reform.

The analysis shows that the reform affected prices and costs via three channels: First, the descriptive evidence shows that the reform had a clear negative effect on prices in all three markets. The strong growth rate of prices declined sharply in 2018, and providers that participated in subsequent procurements increased their prices significantly less than in previous procurements. Second, the reform had a direct impact on costs because most providers with relatively high prices were not offered a contract. Pitkänen et al. (2020) calculated that implementing a similar acceptance rule would have decreased annual costs directly by 1.35 million euros already in 2014. Third, the price effects are strongest in the initially more competitive local physiotherapy markets, where prices decreased by little less than four percent compared to areas with less initial competition. However, this difference between initially more and less competitive areas is not present in the speech and occupational therapy markets. Overall, these results provide empirical evidence that supports the traditional “competition effect” in the studied procurements. The results also highlight the importance of an efficient procurement practice, which includes rule‐based selection criteria and competent procurement officials.

Even though the reform increased price competition and generated cost savings for the procurer, the welfare implications of the reform are ambiguous: Limiting the number of contracted providers forced several patients to switch their incumbent provider for the new contract period. For some patients finding and choosing a new suitable provider can be challenging and may also increase their travel time and costs. On the other hand, the new pool of accepted providers consists mainly of higher quality providers, which may benefit some of the switching patients. Changes in the pool of providers and the resulting search costs for patients are the main disadvantage when repeatedly organized procurements are used in health services that patients receive frequently and for a long time.

Naturally, the study has some limitations as the analysis concentrates solely on the immediate price effects of the procurement reform. In time, providers will likely learn about the risk of not receiving a contract in their service and local markets, which may increase prices in future procurements. Increased competition *for* the market and the new quality‐price scoring rule may also influence providers' quality, especially in the long term. The effects on quality competition *in* the market are also unclear, as a smaller number of providers now compete for patients, many of whom are forced to choose a new provider at the start of the contract period. The reform may also have spillover effects to other markets, such as the procurements organized by Finnish municipalities. For example, increased competition in one market may also increase competition and decrease prices in other markets (Baicker et al., 2013). These long‐term effects and spillovers are a natural direction for a future research agenda.

Maintaining a competitive market environment will be necessary for successful future procurements. After the reform, several providers found themselves without a contract from the largest service purchaser. If these providers do not find new markets and patients, the potential number of bidders may decrease in future procurements. A lower number of contracted providers may also decrease providers' expectations and incentives to participate in future procurements (McCombs & Christianson, 1987). On the other hand, accepted providers can expect a larger number of patients and greater revenue during the contract period, compared to previous procurements where all providers were included in the pool of providers. A potential way to maintain or even increase the overall level of competition, in terms of the number of potential bidders and their capacity, is to increase supply of the required education especially for speech and occupational therapy markets. Providers should also not be highly rewarded based on their previous experiences with the same procurer as it may reduce the willingness of new bidders to participate in procurements (Butler et al., 2020).

## CONFLICT OF INTEREST

The author receives a salary from Kela, which organizes and finances the studied services and procurements. The paper represents the views of the author and does not necessarily represent the official views of Kela.

## Supporting information

Supplementary Material 1

## Data Availability

The data that support the findings of this study are available upon request from the Social Insurance Institution of Finland.

## ENDNOTES

^1^
The procurement data in Jääskeläinen and Tukiainen (2019) does not include data used in this study, but covers procurements of rehabilitation services by the Finnish municipalities. They find that municipalities attract at least as many as and often more competition than procurements made by other larger administrative bodies such as the government.

^2^
Pitkänen and Linnosmaa (2021) show that physiotherapy patients prefer high‐quality providers within a close distance. However, their results indicate that patients who are forced to switch, because their usual provider exits the market, choose their new provider in a limited period of time, leading into poorer choices.

^3^
The number of patients in speech and occupational therapies has increased rapidly because of two main reasons: First, the need for the therapy is recognized earlier than before, and second, a law reform eased access to the services especially for children who are the largest patient group in these services (Heino et al., 2020).

^4^
Kela has centralized its administration over the years: Starting from 59 in 2006, the number of insurance districts declined to 29 in 2010, 25 in 2014, and five in 2018.

^5^
The exceptions are the South Ostrobothnia and Päijät‐Häme districts, which piloted quality‐only procurements with fixed prices (so‐called beauty contests) in physiotherapy service in the 2011–2014 contract period.

^6^
Procurements where multiple winners are selected and their prices are based on their own bids are often called “discriminatory auctions”, because each winner receives a different price (Hoerger & Waters, 1993).

^7^
Even though quality scores are collected in the procurements, Kela has not facilitated patient choice by making the information publicly available to the patients.

^8^
Swedish is an official language in Finland, and individuals are entitled to receive public services in Swedish throughout the country. However, most of the Swedish‐speaking population lives on the southern and western coasts.

^9^
Details on the new procurement practice, including the tighter acceptance criteria and new quality‐price weights, were announced in a public briefing on 22 February 2018. The request for tenders was published on 12 of March and the deadline for submitting a bid was 4 June in 2018. The new contract period started in 1 January 2019.

^10^
Municipalities also finance and organize all three services for patients with less severely disabilities. Compared to speech and occupational therapy firms, the physiotherapy sector has more patients who pay out of pocket or are financed by other organizers such as municipalities, insurance companies or employers.

^11^
In 2019, there were 15 polytechnics providing physiotherapy education, five polytechnics providing occupational therapy education and five universities providing speech therapy education in Finland. The polytechnics accepted a total of 427 new physiotherapy students and 142 new occupational therapy students, whereas the universities accepted 116 new speech therapy students in 2019.

^12^
There are seven different business types in Finland: sole proprietor (around 37% of physiotherapy providers, 63% of speech therapy providers and 45% of occupational therapy providers in 2018), limited liability company (46%, 35% and 48%, respectively), limited partnership (12%, 1% and 2%), general partnership (3%, 0% and 1%), co‐operative (0%, 0% and 1%), association (1%, 1% and 2%) and foundation (1%, 0% and 2%). Associations and foundations are non‐profit organizations, but often operate profitably in the rehabilitation markets to cover their other operating costs.

## References

[hec4485-bib-0001] Asker, J. , & Cantillon, E. (2008). Properties of scoring auctions. The RAND Journal of Economics, 39, 69–85. 10.1111/j.1756-2171.2008.00004.x

[hec4485-bib-0002] Asker, J. , & Cantillon, E. (2010). Procurement when price and quality matter. The RAND Journal of Economics, 41, 1–34.

[hec4485-bib-0003] Baicker, K. , Chernew, M. E. , & Robbins, J. A. (2013). The spillover effects of Medicare managed care: Medicare advantage and hospital utilization. Journal of Health Economics, 32, 1289–1300. 10.1016/j.jhealeco.2013.09.005 24308880 PMC3855665

[hec4485-bib-0004] Bajari, P. , McMillan, R. , & Tadelis, S. (2008). Auctions versus negotiations in procurement: An empirical analysis. The Journal of Law, Economics & Organization, 25, 372–399. 10.1093/jleo/ewn002

[hec4485-bib-0005] Bandiera, O. , Prat, A. , & Valletti, T. (2009). Active and passive waste in government spending: Evidence from a policy experiment. The American Economic Review, 99, 1278–1308. 10.1257/aer.99.4.1278

[hec4485-bib-0006] Barros, P. P. , Brouwer, W. B. F. , Thompson, S. , & Varkevisser, M. (2016). Competition among health care providers: Helpful or harmful? The European Journal of Health Economics, 17, 229–233. 10.1007/s10198-015-0736-3 26467166 PMC4805716

[hec4485-bib-0007] Bergman, M. A. , & Lundberg, S. (2013). Tender evaluation and supplier selection methods in public procurement. Journal of Purchasing and Supply Management, 19, 73–83. 10.1016/j.pursup.2013.02.003

[hec4485-bib-0008] Branco, F. (1997). The design of multidimensional auctions. The RAND Journal of Economics, 28, 63–81. 10.2307/2555940

[hec4485-bib-0009] Bucciol, A. , Camboni, R. , & Valbonesi, P. (2020). Purchasing medical devices: The role of buyer competence and discretion. Journal of Health Economics, 74, 102370. 10.1016/j.jhealeco.2020.102370 33049555 PMC7448819

[hec4485-bib-0010] Bulow, J. , & Klemperer, P. (1996). Auctions versus negotiations. The American Economic Review, 86, 180–194.

[hec4485-bib-0011] Butler, J. V. , Carbone, E. , Conzo, P. , & Spagnolo, G. (2020). Past performance and entry in procurement: An experimental investigation. Journal of Economic Behavior & Organization, 173, 179–195. 10.1016/j.jebo.2020.02.019

[hec4485-bib-0012] Cameron, L. J. (2000). Limiting buyer discretion: Effects on performance and price in long‐term contracts. The American Economic Review, 60, 265–281. 10.1257/aer.90.1.265

[hec4485-bib-0013] Che, Y.‐K. (1993). Design competition through multidimensional auctions. The RAND Journal of Economics, 24, 668–680. 10.2307/2555752

[hec4485-bib-0014] Cooper, Z. , Gibbons, S. , Jones, S. , & McGuire, A. (2011). Does hospital competition save lives? Evidence from the English NHS patient choice reforms. Economic Journal, 121, 228–F260. 10.1111/j.1468-0297.2011.02449.x PMC4373154 25821239

[hec4485-bib-0015] Coviello, D. , Guglielmo, A. , & Spagnolo, G. (2018). The effect of discretion on procurement performance. Management Science, 64, 715–738. 10.1287/mnsc.2016.2628

[hec4485-bib-0016] Decarolis, F. , Giuffrida, L. M. , Iossa, E. , Mollisi, V. , & Spagnolo, G. (2020). Bureaucratic competence and procurement outcomes. Journal of Law, Economics, and Organization, 36, 537–597. 10.1093/jleo/ewaa004

[hec4485-bib-0017] Dietrichson, J. , Ellegård, L. M. , & Kjellsson, G. (2020). Patient choice, entry, and the quality of primary care: Evidence from Swedish reforms. Health Economics, 29, 716–730. 10.1002/hec.4015 32187777

[hec4485-bib-0018] Dranove, D. , & Ody, C. (2015). Evolving measures of provider market power. American Journal of Health Economics, 2, 145–160. 10.1162/AJHE_a_00039

[hec4485-bib-0019] European Commission . (2020). *Public procurement*. Retrieved April 24, 2020, from https://ec.europa.eu/growth/single‐market/public‐procurement_en

[hec4485-bib-0020] Expert panel on effective ways of investing in health (EXPH) . (2021). *Public procurement in healthcare systems*. Retrieved September 20, 2021, from https://ec.europa.eu/health/sites/default/files/expert_panel/docs/027_public_proc_healthcare_sys_en.pdf

[hec4485-bib-0021] Ferraresi, M. , Gucciardi, G. , & Rizzo, L. (2021). Savings from public procurement centralization in the healthcare system. European Journal of Political Economy, 66, 101963. 10.1016/j.ejpoleco.2020.101963

[hec4485-bib-0022] Forder, J. , & Allan, S. (2014). The impact of competition on quality and prices in the English care homes market. Journal of Health Economics, 34, 73–83. 10.1016/j.jhealeco.2013.11.010 24487075 PMC4155013

[hec4485-bib-0023] Fricke, H. (2017). Identification based on difference‐in‐differences approaches with multiple treatments. Oxford Bulletin of Economics & Statistics, 79, 426–433. 10.1111/obes.12178

[hec4485-bib-0024] Gaynor, M. , Moreno‐Serra, R. , & Propper, C. (2013). Death by market power: Reform, competition and patient outcomes in the British National Health Service. American Economic Journal: Economic Policy, 5, 134–166. 10.1257/pol.5.4.134

[hec4485-bib-0025] Heino, P. , Mäkinen, J. , & Seppänen‐Järvelä, R. (2020). Vaativan lääkinnällisen kuntoutuksen lainmuutoksen vaikutus kuntoutuksen kohdentumiseen. Rekisteritutkimus vuosien 2014, 2016 ja 2017 kuntoutuspäätöksistä. Kela. Sosiaali‐ ja terveysturvan raportteja 23.

[hec4485-bib-0026] Hoerger, T. J. , & Waters, T. M. (1993). Competitive bidding for Medicare services. Medical Care, 31, 879–897. 10.1097/00005650-199310000-00003 8412391

[hec4485-bib-0027] Hong, H. , & Shum, M. (2002). Increasing competition and the winner's curse: Evidence from procurement. The Review of Economic Studies, 69, 871–898. 10.1111/1467-937X.00229

[hec4485-bib-0028] Hyytinen, A. , Lundberg, S. , & Toivanen, O. (2018). Design of public procurement auctions: Evidence from cleaning contracts. The RAND Journal of Economics, 49, 398–426. 10.1111/1756-2171.12232

[hec4485-bib-0029] Jääskeläinen, J. , & Tukiainen, J. (2019). Anatomy of public procurement. VATT. *Working Paper 129*.

[hec4485-bib-0030] Li, T. , & Zheng, X. (2009). Entry and competition effects in first‐price auctions: Theory and evidence from procurement auctions. The Review of Economic Studies, 76, 1397–1429. 10.1111/j.1467-937X.2009.00558.x

[hec4485-bib-0031] McCombs, J. S. , & Christianson, J. B. (1987). Applying competitive bidding to health care. Journal of Health Politics, Policy and Law, 12, 703–722. 10.1215/03616878-12-4-703 3323294

[hec4485-bib-0032] Mukamel, D. B. , & Spector, W. D. (2002). The competitive nature of the nursing home industry: Price mark ups and demand elasticities. Applied Economics, 34, 413–420. 10.1080/00036840110044199

[hec4485-bib-0033] Nyman, J. A. (1994). The effects of market concentration and excess demand on the price of nursing home care. The Journal of Industrial Economics, 42, 193–204. 10.2307/2950490

[hec4485-bib-0034] Onur, I. , & Tas, B. K. O. (2019). Optimal bidder participation in public procurement auctions. International Tax and Public Finance, 26, 595–617. 10.1007/s10797-018-9515-2

[hec4485-bib-0035] Pekola, P. , Linnosmaa, I. , & Mikkola, H. (2017). Does competition have an effect on price and quality in physiotherapy? Health Economics, 26, 1278–1290. 10.1002/hec.3402 27619843

[hec4485-bib-0036] Pinkse, J. , & Tan, G. (2005). The affiliation effect in first‐price auctions. Econometrica, 73, 263–277. 10.1111/j.1468-0262.2005.00571.x

[hec4485-bib-0037] Pitkänen, V. , Jauhiainen, S. , & Linnosmaa, I. (2020). Low risk, high reward? Repeated competitive biddings with multiple winners in health care. The European Journal of Health Economics, 21, 483–500. 10.1007/s10198-019-01143-1 31902025 PMC7214509

[hec4485-bib-0038] Pitkänen, V. , & Linnosmaa, I. (2021). Choice, quality and patients’ experience: Evidence from a Finnish physiotherapy service. International Journal of Health Economics and Management, 21, 229–245. 10.1007/s10754-020-09293-z 33469804 PMC8192355

[hec4485-bib-0039] Poutvaara, P. , & Jordahl, H. (2020). Public sector outsourcing (Vol. 65). IZA World of Labor. 10.15185/izawol.65.v2

[hec4485-bib-0040] Propper, C. , Burgess, S. , & Gossage, D. (2008). Competition and quality: Evidence from the NHS internal market 1991–9. The Economic Journal, 118, 138–170. 10.1111/j.1468-0297.2007.02107.x

[hec4485-bib-0041] Roos, A.‐F. , O’Donnell, O. , Schut, F. T. , Van Doorslaer, E. , Van Gestel, R. , & Varkevisser, M. (2020). Does price deregulation in a competitive hospital market damage quality? Journal of Health Economics, 72, 102328. 10.1016/j.jhealeco.2020.102328 32599157

[hec4485-bib-0042] Scheffler, P. , Schiele, H. , & Horn, P. (2016). How to measure competition? The role of price dispersion in B2B supply markets. International Journal of Procurement Management, 9, 568–586. 10.1504/IJPM.2016.078692

[hec4485-bib-0043] Song, Z. , Landrum, M. B. , & Chernew, M. E. (2012). Competitive bidding in Medicare: Who benefits from competition? American Journal of Managed Care, 18, 546–552. 23009305 PMC3519284

[hec4485-bib-0044] Song, Z. , Landrum, M. B. , & Chernew, M. E. (2013). Competitive biddings in Medicare Advantage: Effect of benchmark changes on plan bids. Journal of Health Economics, 32, 1301–1312. 10.1016/j.jhealeco.2013.09.004 24308881 PMC3893317

[hec4485-bib-0045] Suomen Virallinen Tilasto (SVT) . (2019). Kuluttajahintaindeksi. ISSN=1796‐3524. Tilastokeskus. Retrieved December 12, 2019, from http://www.stat.fi/til/khi/index.html

[hec4485-bib-0046] Tas, B. K. O. (2020). Effect of public procurement regulation on competition and cost‐effectiveness. Journal of Regulatory Economics, 58, 59–77. 10.1007/s11149-020-09409-w

[hec4485-bib-0047] Tay, A. (2003). Assessing competition in hospital care markets: The importance of accounting for quality differentiation. The RAND Journal of Economics, 34, 786–814. 10.2307/1593788 15025031

[hec4485-bib-0048] Van Eijkel, R. , Kattenberg, M. , & Van der Torre, A. (2018). Competition and pricing behavior in long‐term care markets: Evidence from the market for assistance in daily housekeeping activities. CPB. *(Discussion Paper 373)*.

[hec4485-bib-0049] Wilson, R. (1977). A bidding model of perfect competition. The Review of Economic Studies, 44, 511–518. 10.2307/2296904

