# Supplementary material for: Competition and efficiency in repeated procurements: Lessons from the Finnish rehabilitation markets
Source: Health Econ. 2022 Feb 20;31(5):820–35. doi: 10.1002/hec.4485 (PMC9304294; doi:10.1002/hec.4485)
Supplement: Supplementary file 1 — Supplementary Material 1 [file HEC-31-820-s001.pdf]

Appendix

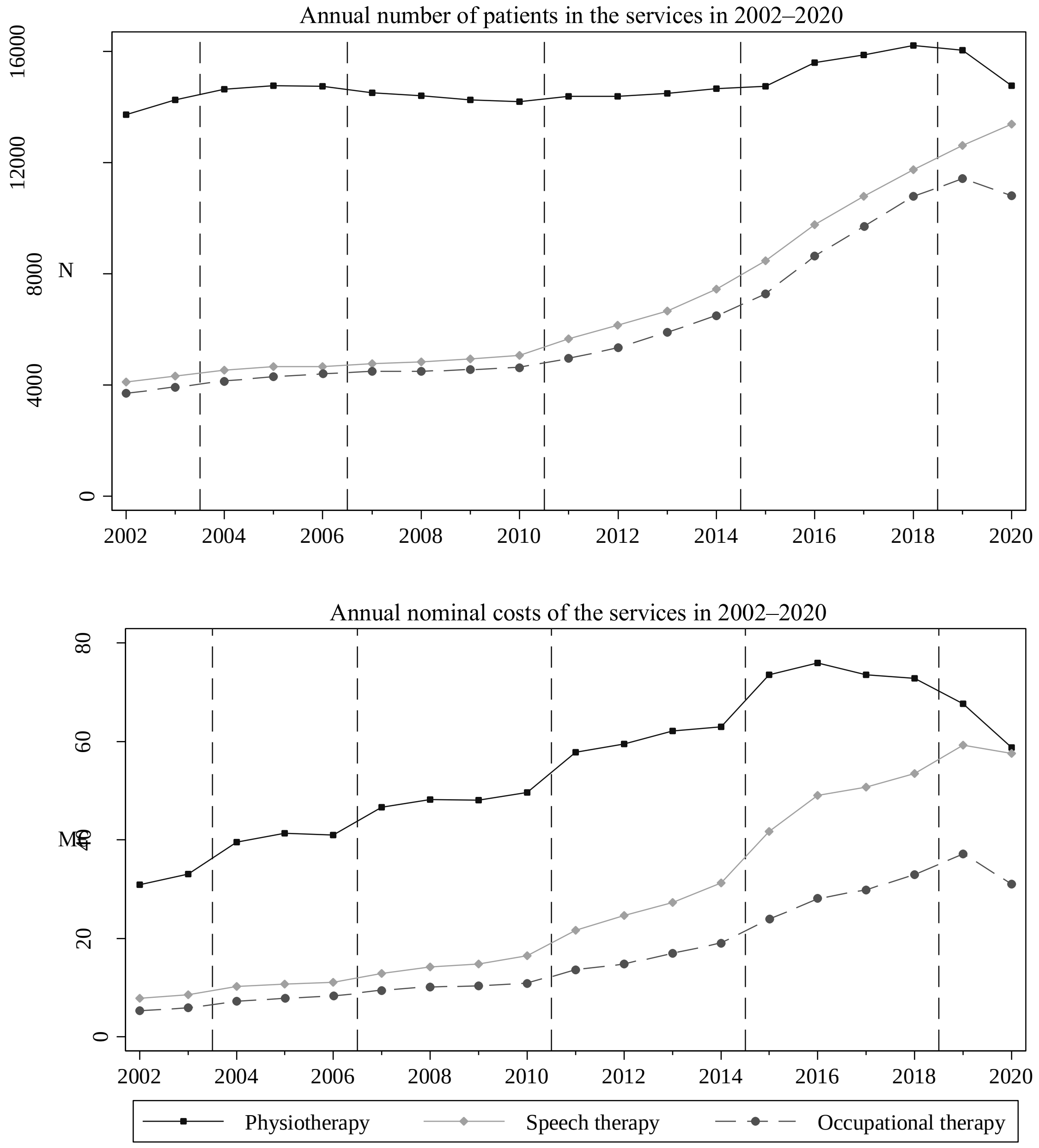

Figure A1. Annual number of patients and costs in 2002–2020.

Notes: Vertical dashed lines present the contract periods. Presented costs are nominal. The costs include service costs and travel costs by providers, but do not include travel costs by patients.

Table A1. Descriptive statistics of physiotherapy providers.

	Accepted providers					Rejected providers				
Variable	N	Mean	SD	Min	Max	N	Mean	SD	Min	Max
2003										
Price (€)	1364	33.41	4.52	20	55	–	–	–	–	–
2006										
Price (€)	1284	39.24	5.73	21	64.5	11	41.95	8.00	30	55
Quality	1284	67.09	12.06	28	103	11	58.18	16.58	24	78
Capacity	530	17.50	20.20	1	200	0	0	0	0	6
ΔPrice (€)	1058	5.54	3.44	–21	32.9	5	6.10	6.19	2	17
2010										
Price (€)	1197	47.53	7.62	28	99	7	54.07	10.22	35	68.5
Quality	1197	80.47	13.62	31	104	7	68.29	16.01	44	84
Capacity	1197	33.78	43.37	1	420	7	40.14	67.95	5	192
ΔPrice (€)	1018	8.12	5.05	–27.5	32	2	10	4.24	7	13
2014										
Price (€)	1159	57.83	9.43	34	102.5	28	70.38	16.80	45	116
Quality	1159	37.41	7.45	9	55	28	26.57	7.39	14	43
Capacity	1159	41.00	53.73	1	450	28	13.64	13.83	2	70
ΔPrice (€)	930	10.08	5.79	–16	54.5	11	15.62	12.80	1.58	40
2018										
Price (€)	793	60.52	8.38	35	100	295	68.57	7.73	50	105
Quality	793	44.85	9.00	0	50	295	38.49	14.66	0	50
Capacity	793	44.12	71.80	1	1000	295	41.35	70.91	1	760
ΔPrice (€)	632	3.42	5.55	–35	24	184	5.63	6.49	-20	25

Notes: Quality scores are not comparable between the competitive biddings.

Table A2. Descriptive statistics of speech therapy providers.

	Accepted providers					Rejected providers				
Variable	N	Mean	SD	Min	Max	N	Mean	SD	Min	Max
2003										
Price (€)	371	54.88	6.25	36	80	–	–	–	–	–
2006										
Price (€)	480	66.15	8.60	42	100	6	77.50	4.18	70	80
Quality	258	65.20	11.48	34	102	6	54.17	12.25	40	71
Capacity	130	15.12	9.21	1	50	3	68.33	54.85	5	100
ΔPrice (€)	323	9.31	5.16	0	32.8	0	0	0	0	0
2010										
Price (€)	481	82.24	13.03	54	150	7	70.29	10.92	60	91
Quality	481	74.92	14.33	24	101	7	50.29	4.82	43	57
Capacity	481	17.65	14.12	1	200	7	29.14	27.61	8	90
ΔPrice (€)	352	16.26	11.19	–12	70.85	1	9	0	9	9
2014										
Price (€)	577	101.15	14.21	58	175	4	111.00	20.35	95	140
Quality	577	32.15	8.58	0	53	4	33.00	8.16	23	43
Capacity	577	20.78	22.01	1	180	4	28.00	29.09	7	70
ΔPrice (€)	415	17.01	10.97	–19	70	0	0	0	0	0
2018										
Price (€)	580	111.82	13.73	74.95	160.14	9	115.76	11.44	99	132
Quality	580	26.55	13.58	0	40	9	28.78	12.16	5	40
Capacity	580	23.86	58.66	1	800	9	13.44	6.25	4	20
ΔPrice (€)	402	10.14	10.54	–20	47	6	8.13	12.99	0	33

Notes: Quality scores are not comparable between the competitive biddings.

Table A3. Descriptive statistics of occupational therapy providers.

	Accepted providers					Rejected providers				
Variable	N	Mean	SD	Min	Max	N	Mean	SD	Min	Max
2003										
Price (€)	279	45.05	6.79	25.5	84	–	–	–	–	–
2006										
Price (€)	325	52.76	8.06	27	80	5	69.80	22.81	29	80
Quality	177	65.63	14.37	31	95	5	54.20	6.76	49	63
Capacity	55	16.45	9.80	1	50	2	80	28.28	60	100
ΔPrice (€)	230	7.16	4.88	–12.75	30	0	0	0	0	0
2010										
Price (€)	388	63.48	8.08	42	86.8	8	59.16	7.57	46	68.5
Quality	388	78.97	14.06	36	105	8	60.13	4.97	53	68
Capacity	388	27.10	38.06	3	340	8	20	10.45	10	40
ΔPrice (€)	254	11.04	6.28	–11.5	30	2	–4.35	10.11	–11.5	2.80
2014										
Price (€)	481	74.31	10.07	40	110	4	89.04	21.42	70	110
Quality	481	35.58	8.09	11	54	4	24.75	11.03	17	41
Capacity	481	33.61	42.63	0	400	4	29.75	17.23	9	50
ΔPrice (€)	314	10.84	6.54	–17	34	0	0	0	0	0
2018										
Price (€)	465	77.28	8.93	49.9	105	52	92.28	24.53	71	249
Quality	465	32.15	11.33	0	40	52	17.04	16.78	0	40
Capacity	465	33.76	48.50	1	500	52	26.87	33.08	2	220
ΔPrice (€)	308	4.28	6.65	–25	29.26	22	9.88	10.50	–5	34

Notes: Quality scores are not comparable between the competitive biddings.

Table A4. Additional provider characteristics in the 2018 procurement.

Variable	Mean	SD	Min	Max
Physiotherapy				
Premises	0.91	0.28	0	1
Capacity	43.37	71.54	1	1000
Therapists	3.24	3.46	1	25
Chain	0.35	0.48	0	1
N	1088			
Speech therapy				
Premises	0.70	0.46	0	1
Capacity	23.71	58.23	1	800
Therapists	1.97	3.79	1	59
Chain	0.29	0.45	0	1
N	589			
Occupational therapy				
Premises	0.83	0.37	0	1
Capacity	33.07	47.20	1	500
Therapists	2.32	2.37	1	21
Chain	0.40	0.49	1	0
N	517			

Notes: Bidders with the same business identity code are calculated as chains.

Table A5. Number of providers in the treatment, control and excluded groups.

	Treatment	Control	Excluded
Physiotherapy			
2003	588	382	394
2006	559	338	398
2010	533	328	343
2014	552	271	364
2018	485	262	341
Speech therapy			
2003	165	42	164
2006	214	49	223
2010	208	41	239
2014	271	43	267
2018	273	56	260
Occupational therapy			
2003	170	49	60
2006	191	55	84
2010	245	46	105
2014	311	54	120
2018	333	64	120

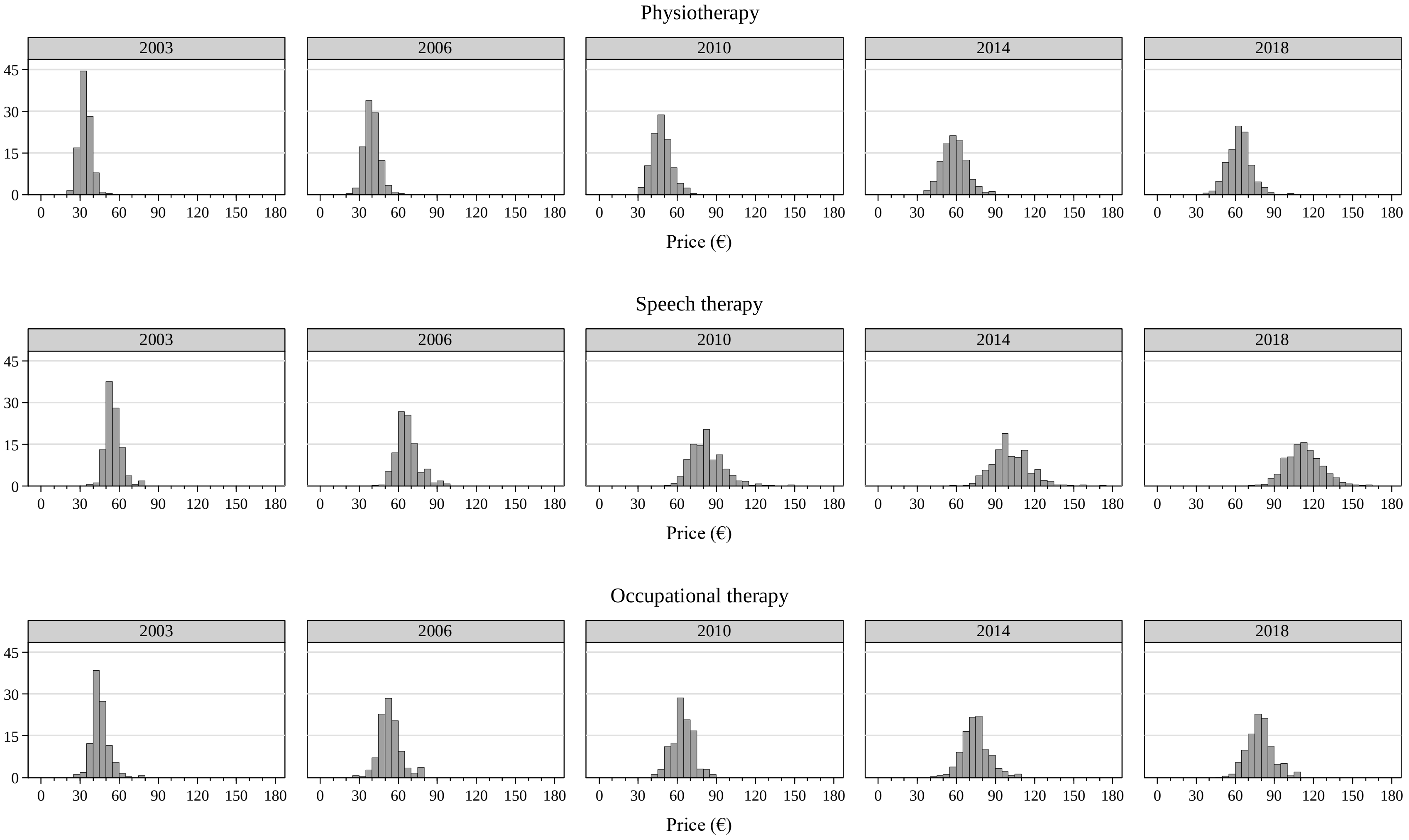

Figure A2. Prices in the procurements.

Notes: One outlier bidder in occupational therapy (249€ in 2018) has been excluded from the figure.

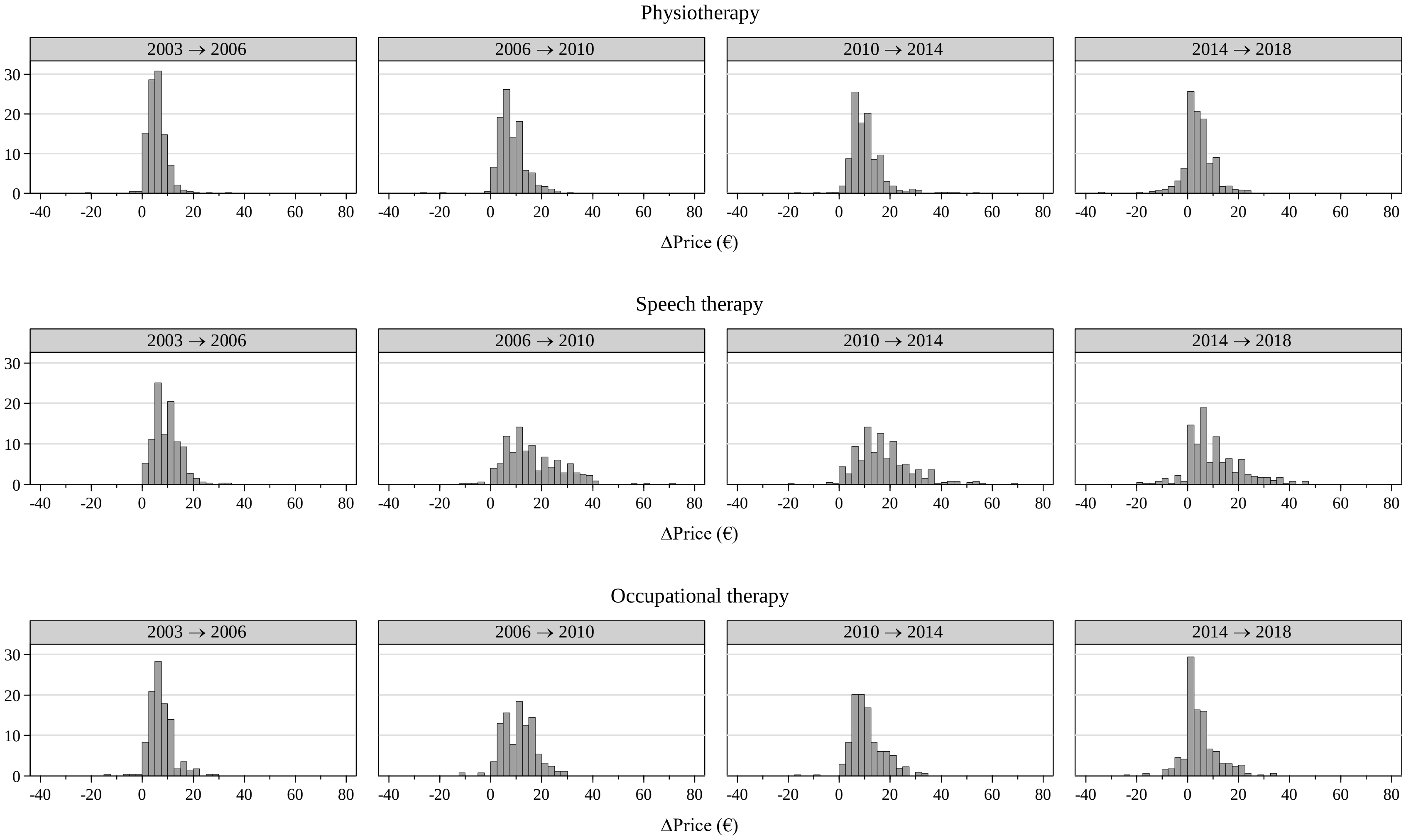

Figure A3. Price changes in the procurements.

Table A6. Robustness checks.

	Physiotherapy			Speech therapy			Occupational therapy		
	(1)	(2)	(3)	(1)	(2)	(3)	(1)	(2)	(3)
DID	0.006	–0.037***	–0.034**	–0.0001	–0.027	–0.023	–0.016	–0.026	–0.021
	(0.010)	(0.009)	(0.010)	(0.047)	(0.020)	(0.022)	(0.026)	(0.023)	(0.022)
Quality			0.004**			0.003			0.003
			(0.001)			(0.002)			(0.002)
Capacity			–0.000			–0.0003			–0.0001
			(0.000)			(0.0002)			(0.0002)
Year FE	Yes	Yes	Yes	Yes	Yes	Yes	Yes	Yes	Yes
District FE	Yes	Yes	Yes	Yes	Yes	Yes	Yes	Yes	Yes
Provider FE	Yes	Yes	Yes	Yes	Yes	Yes	Yes	Yes	Yes
Observations	3551	6138	2431	1033	2515	892	1121	2007	1053
Providers	1369	2263	1156	477	1054	490	515	886	579
R^2	0.645	0.723	0.397	0.377	0.750	0.445	0.365	0.635	0.244

Notes: Dependent variable: Ln(Price). Compared to the main regressions (Table 3), Model 1 estimates a placebo treatment for year 2010; providers that are located in areas with prior rejections are included in Model 2; and Model 3 adds controls for providers' quality and capacity using data from 2010, 2014 and 2018.
*p<0.05; **p<0.01; ***p<0.001.

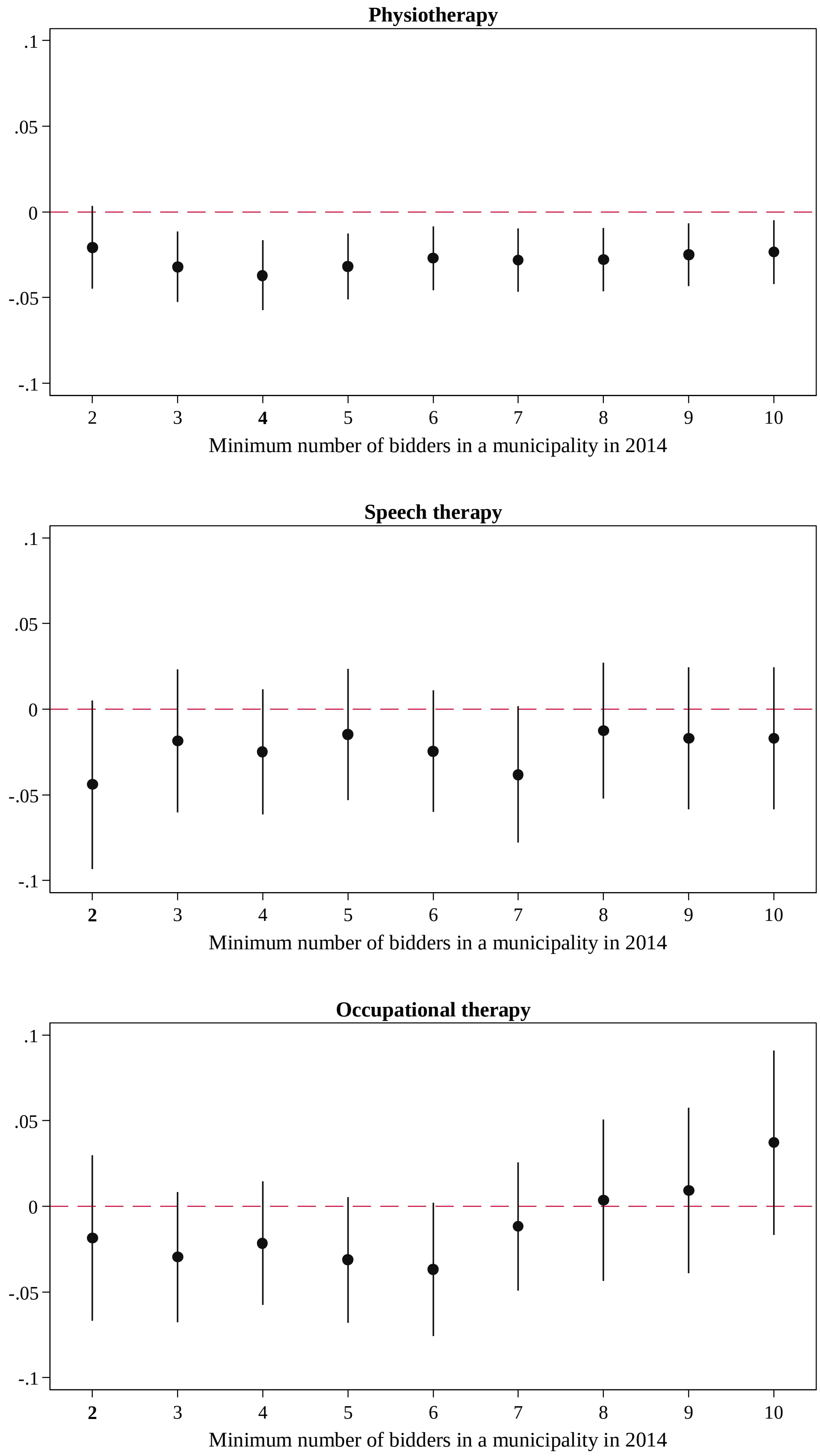

Figure A4. Price effects using different thresholds for the treatment group.
